# Competition in the presence of aging: dominance, coexistence, and alternation between states

**DOI:** 10.1038/srep21128

**Published:** 2016-02-16

**Authors:** Toni Pérez, Konstantin Klemm, Víctor M. Eguíluz

**Affiliations:** 1 Instituto de Física Interdisciplinar y Sistemas Complejos (IFISC), Palma de Mallorca, E-07122, Spain; 2 School of Science and Technology, Nazarbayev University, Astana, 010000, Kazakhstan; 3 Bioinformatics, Institute of Computer Science, University Leipzig, Leipzig, 04107, Germany; 4 Bioinformatics and Computational Biology, University of Vienna, Vienna, 1090, Austria; 5 Theoretical Chemistry, University of Vienna, Vienna, 1090, Austria

## Abstract

We study the stochastic dynamics of coupled states with transition probabilities depending on local persistence, this is, the time since a state has changed. When the system has a preference to adopt older states the system orders quickly due to the dominance of old states. When preference for new states prevails, the system can show coexistence of states or synchronized collective behavior resulting in long ordering times. In this case, the magnetization of the system oscillates around zero. Finally we discuss a potential application in social systems.

Models of two states are commonly used in physics as a tool to study the emergence of collective behavior in systems from spin interaction to opinion dynamics^1^,^2^,^3^. In the adoption of traits^4^,^5^,^6^,^7^ different aspects have been studied including the relevance of the interaction topology^8^,^9^,^10^, social influence^11^,^12^, and mass media^13^,^14^,^15^. When accounting for opinion dynamics, the majority of models are based on decision rules that consider a fraction of the surrounding states, e.g., voter model^16^, threshold model^17^, majority rule^18^, or Sznajd model^19^.

The timing of the interactions can also affect the behavior of the system at least by two ways: the precise sequence of interactions and by the aging of states. For example, in epidemic spreading and diffusion, the temporal sequence of interactions can slow down the spreading process^20^,^21^,^22^,^23^; in ordering dynamics, state-dependent updates can have a qualitative impact on the mean time to order^24^,^25^,^26^,^27^,^28^,^29^. Aging in physical systems refers to the persistence time, that is, the time spent in a given state, and affects the response of the system to an external field or perturbation^30^,^31^. In social systems, when individuals make choices they usually rely on their own past experience or memory^32^,^33^,^34^. While conservative groups tend to hold ideas in an unaltered form for a long time, progressive individuals embrace new opinions, ideas, or a technology and disseminate them with more enthusiasm^35^,^36^. In the competition between new and old information, although new information is more valuable for exploring and spatial searching^37^, adopting older strategies can promote cooperation and group success^38^. In the emergence of cooperation, the persistence time in the learning of strategies in the spatial prisoner’s dilemma enhances cooperation and leads to heterogeneous distributions of persistence times^39^, and generates cyclic dominance of strategies^40^. In an evolutionary context, aging at the speciation events has been proposed as a mechanism to explain the shape of evolutionary trees^41^. Another example of the impact of aging can be found in the citation networks, where the age of the nodes has a crucial effect in the dynamic of growing of the network^42^. Finally, in epidemic spreading persistence has been introduced in the modeling of the mobility of the agents^43^,^44^ and as latency periods in the dynamics of infection^21^,^45^.

Here we analyze how the tendency of particles towards the adoption of established vs. novel states influences the macroscopic dynamics and the ordering process. We tackle this problem by considering a model with randomly chosen pairwise interactions. The adoption of one of the particles’ states depends on their relative age or persistence time, that is, the time span each particle has been in its current state. Throughout this work we will disregard the lifetime of the particle and we will use the terms young and old to describe, in a pairwise interaction, the particle with the smallest and largest persistence time, respectively.

## Results

The system is composed by *N* particles whose dynamic is defined as follows: each particle has a state *L* that can be up (↑) or down (↓) with age young (*Y*) or old (*Z*). Thus, particles can be in four states *Y*^↑^, *Y*^↓^, *Z*^↑^, and *Z*^↓^. Young states turn into old states at a rate that we set to *r* = 1. Then, there are interactions of randomly paired particles: i) for young *i* and old *j* of opposite states, *i* adopts the state of *j* with probability 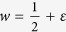, otherwise (with probability 1 − *w*), *j* adopts the state of *i*; ii) for pairs of particles with the same age and different states, each particle has probability 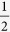 of convincing the other; iii) for pairs of particles with the same state, nothing happens. When a particle adopts a state, the persistence time goes to the young age of the adopted state. Neglecting correlations, the expectation values of state fractions evolve according to


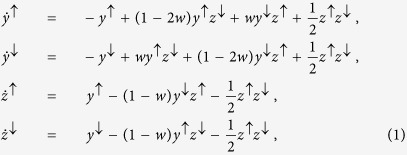


with the normalization *y*^↑^ + *y*^↓^ + *z*^↑^ + *z*^↓^ = 1. Here we use *y*^↑↓^ and *z*^↑↓^ to refer to the fraction of the corresponding states occupied by the particles. The parameter *ε* corresponds to the persuasiveness of the particle: *ε* > 0 means that particles with older states are more persuasive, while for *ε* < 0, particles with younger states are more persuasive.

**Figure 1:**
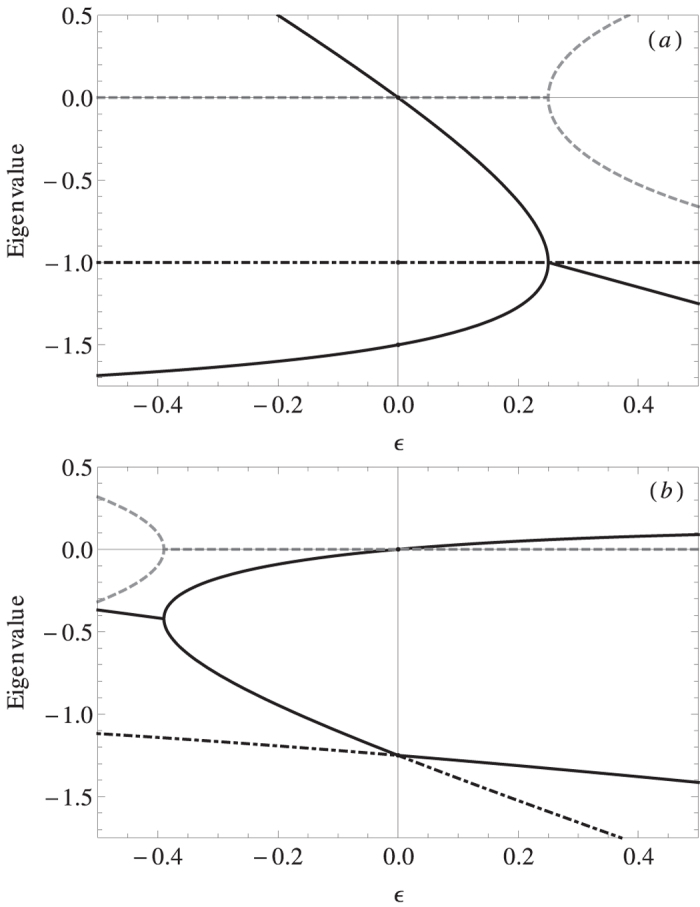
Jacobian eigenvalues of the stationary solutions of Eq. (1) as a function of persuasiveness *ε* for: (**a**) the homogeneous solutions *S*_1_ and *S*_2_ having the same eigenvalues, and (**b**) the solution *S*_3_. Black solid (gray dashed) lines represent real (imaginary) parts of the two complex conjugate eigenvalues. Dotted-dashed black line represents the third eigenvalue (real). The fourth eigenvalue (not shown) for the eigenspace in (1, 1, 1, 1) direction is zero due to conservation of normalization.

The system presents three stationary solutions in the relevant range of all four variables being non-negative. Two fixed points are the homogeneous solutions *S*_1_ having *z*^↓^ = 1 and *S*_2_ having *z*^↑^ = 1. Here either all states are down (*S*_1_) or all are up (*S*_2_) and old. Figure 1(a) shows the Jacobian eigenvalues for these two solutions as a function of *ε*. These homogeneous solutions are stable if *ε* > 0. Non-zero imaginary parts of two eigenvalues are obtained for *ε* > 1/4. The third fixed point *S*_3_ is an up-down-symmetric solution with values 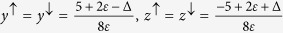 where 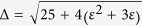. As shown in Fig. 1(b), *S*_3_ is stable if *ε* < 0, thus complementary to the stability of the homogeneous solutions. A transition from zero to non-zero imaginary parts of two eigenvalues occurs when *ε* falls below approximately −0.39. In this regime of strongly negative *ε*, the system oscillates when relaxing from a perturbation out of the symmetric fixed point solution *S*_3_. This stability scenario is qualitatively maintained when *r* changes. As *r* → 0, the point at which the non-zero imaginary part of the eigenvalues appears shifts towards *ε* = 0.

### Model with continuous ages

We now move from two ages to a continuous age space and introduce an age dependent probability. The model is described as follows. Particles can be in one of the two states, up or down. The state of each particle has associated a persistence time defined as: *τ*_*i*_ = *t* − *t*_*i*_, where *t* is the current time and *t*_*i*_ is the time when the current state of particle *i* was acquired. The system evolves by randomly selecting a pair of particles that, if they are in different states, with probability *p*_*i* → *j*_ particle *j* adopts the state of particle *i*, otherwise (with probability 1 − *p*_*i* → *j*_), *i* copies the state of *j*. If the particles are already in the same state, no change is applied. After *N* updates, time *t* is increased to *t* + 1. The probability *p*_*i* → *j*_ depends on the persistence times *τ*_*i*_ and *τ*_*j*_ as


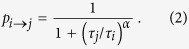


Initially, each particle has randomly assigned one of the two states and the initial persistence times *τ*_*i*_ are uniformly distributed proportionally to the system size *N*. We consider random mixing where each particle is allowed to interact with any other particle. The case *α* = 0 corresponds to an updating probability of *p*_*i* → *j*_ = 0.5 which leads to voter model dynamics^16^. Large positive values of the exponent (*α* → ∞) correspond to situations in which the particle with the initial oldest state, i.e., the initial largest persistence time, is imposing her state. For large negative values (*α* → −∞), the state of the particle with the current youngest state, i.e., the shortest persistence time, is imposed.

We first examine the absorbing states of the system. For *α* → ∞ the system ends up in the oldest state while for values of *α* ∈ (−∞, 0] the system adopts any of the two states with equal probability. For *α* ∈ (0, ∞) the probability that the system adopts the initial oldest state grows with increasing *α* and it tends to 1/2 when *N* increases.

**Figure 2:**
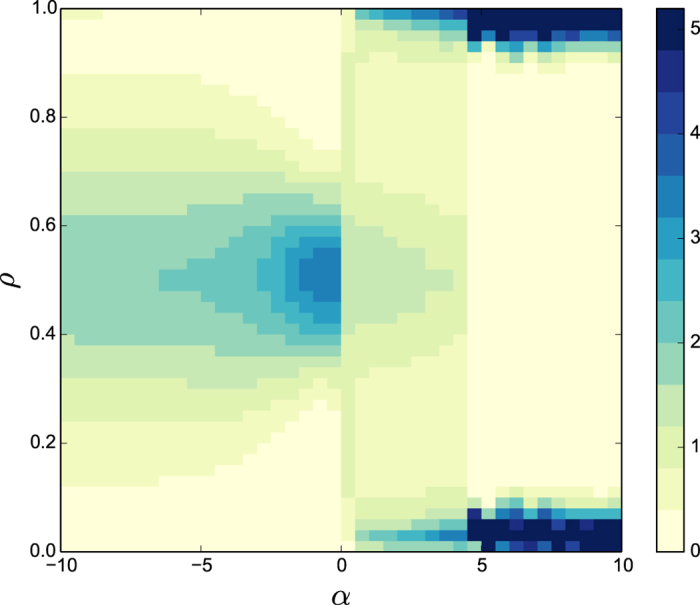
Probability density function (codified as a color map) of the dependence of the states of the system *ρ* with *α*. The probability density function of the states is averaged over 10^5^ realizations. The system size is fixed to *N* = 100.

We define the magnetization of the system 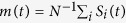 where *S*_*i*_(*t*) takes value +1 if the particle *i* is at time *t* in state up and −1 if the particle is in state down. The state of the system at time *t* is defined as *ρ*(*t*) = 〈(*m*(*t*) + 1)/2〉, where 〈⋅〉 denotes average over independent realizations. From *ρ*(*t*), the probability density function of the system is computed during the simulation period of *T* = 10^7^ Monte Carlo steps. Figure 2 shows the probability distribution function of the states of the system *ρ* as a function of *α*. For *α* = 0, the distribution of states is homogeneous corresponding to a uniform distribution of the states. For *α* negative but close to zero, the dynamic is concentrated around *ρ* = 0.5, which corresponds to a configuration where the particles alternate between any of the two states. This situation changes gradually to a more homogeneous distribution of states as *α* becomes more negative. For *α* > 0, the states are concentrated close to 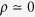 and 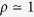 showing that the system eventually orders in one of the two states (the presence of the two peaks is due to the random initial conditions).

**Figure 3:**
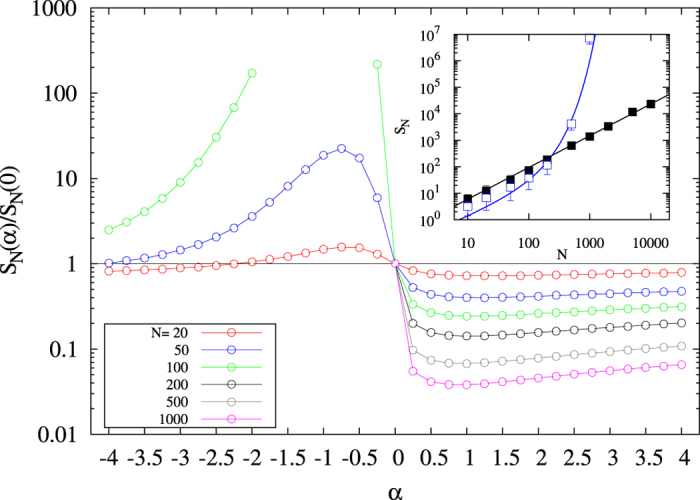
Rescaled ordering time *S*_*N*_(*α*)/*S*_*N*_(0) versus *α* for different system sizes. Open symbols stand for the median of the ordering time normalized to the median of the ordering time at *α* = 0. The horizontal black line indicates ordering time equal to that of the voter model without age influence (*α* = 0) and it has been added for visualization purposes. Inset: Scaling of the median of *S*_*N*_(*α*) in the limits *α* → ∞ (solid symbols) and *α* → −∞ (open symbols). Solid lines fit respectively *S*_*N*_(+∞) ~ *N*^*γ*^ with *γ* = 1.2 (black) and *S*_*N*_(−∞) ~ *N* exp(*bN*) with *b* = 0.009 (blue). Ordering times in the region *α* < 0 for *N* ≥ 100 are not shown because they are computationally very expensive in time.

Figure 3 shows the ordering time *S*_*N*_(*α*), i.e., the time that the system needs to reach a final state where all the particles have the same state, computed as the median of the distribution of ordering times from different simulations and rescaled to the value *S*_*N*_(*α* = 0). *S*_*N*_(0) increases linearly with the *N* as it does for the voter model^9^,^46^. For values *α* > 0, *S*_*N*_(*α*) gets smaller than *S*_*N*_(0) implying that the system orders faster than in the voter model. There is a transition when *α* crosses zero. For values 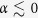, *S*_*N*_(*α*) increases very fast with *N*. This is in agreement with the observed dynamics around *α* = −1 (see Fig. 2). The inset of Fig. 3 shows the scaling with system size of *S*_*N*_(*α*) in the limits: i) *α* = +∞ corresponding to the case where, when confronting two states, the oldest state always induces the change, and ii) *α* =−∞ where the youngest state always induces the change. For *α* = +∞ the ordering time scales as *S*_*N*_ ~ *N*^*γ*^ with the exponent *γ* = 1.2. In the other limit, for *α* = −∞, the ordering time scales as *S*_*N*_ ~ *N* exp(*bN*) with *b* = 0.009.

**Figure 4:**
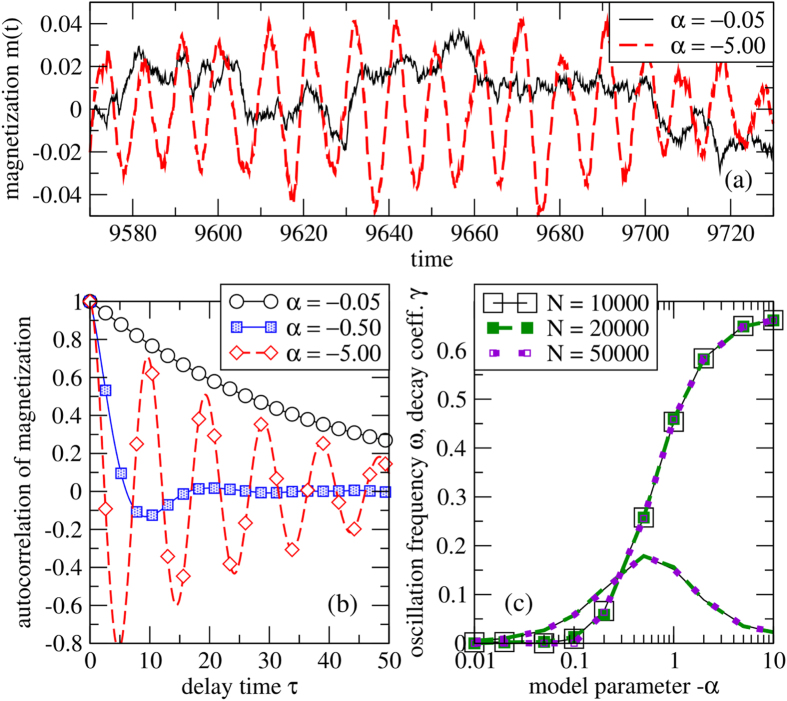
Oscillations and decay of correlations for the finite-size model in the regime *α* < 0. (**a**) Excerpts from time series of the magnetization for two different values of *α*, system size *N* = 50000. (**b**) Autocorrelation functions from magnetization time series of length *T* = 10^5^, system size *N* = 50000. (**c**) Oscillation frequency *ω* (curves with symbols) and decay coefficients *γ* (no symbols) extracted from time series under different values of *α* and *N*. Curves for different system sizes *N* are almost indistinguishable. From the autocorrelation function *A*(*τ*), frequency *ω* and decay coefficient *γ* are obtained from a least squares fit of the functional form *A*_fit_(*t*) = exp(−*γt*)cos(*ωt*) in the range *τ* ∈ [0, 100].

In the regime *α* < 0, what is the behaviour of the system during the long ordering times? Figure 4(a) shows time series of the magnetization of the system *m*(*t*). For *α* negative and sufficiently far from zero, the magnetization oscillates around zero. Figure 4(b) provides further analysis by the autocorrelation functions of the magnetization time series. The onset of oscillations is observed when *α* passes a value around −0.5 from above. Figure 4(c) shows the frequency *ω* and decay constant *γ* extracted from least squares fits to the autocorrelation functions. These values do not exhibit significant dependence on system size. The decay constant is maximum at the onset of oscillations, i.e. where the frequency *ω* becomes non-zero. Both the onset of oscillations and the decay behaviour are captured by the basic model, cf. Fig. 1(b). At the transition to non-zero imaginary parts (oscillations), the stability of the symmetric fixed point solution (*S*_3_) is maximal, meaning that perturbations decay fastest.

To understand further the dynamics around *α* = 0 we define a quantity called the convincingness *z*. Let *S*^+^, *S*^−^ ∈ [*N*] be the two sets of particles with equal states within each set and different states across sets. We define the *convincingness* of *S*^+^ vs. *S*^−^ as the probability *z* that the interaction of a uniformly random pair of an *S*^+^ particle and an *S*^−^ particle leads to adoption of the *S*^+^ state,


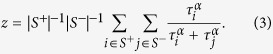


**Figure 5:**
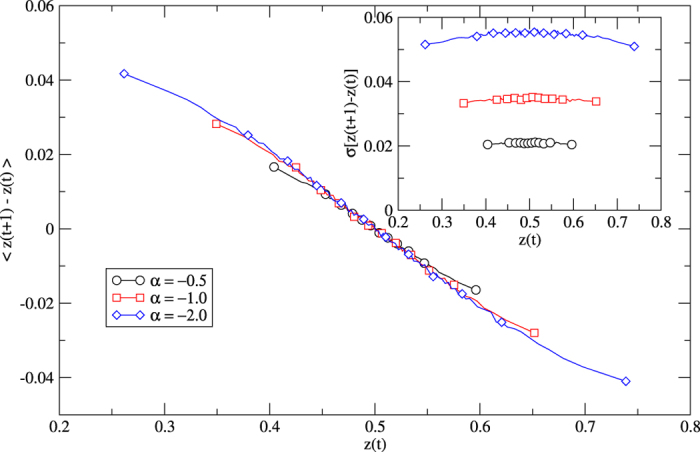
Average restoring force on convincingness *z* following Hooke’s law over a wide range. Data pairs (*z*(*t*), *z*(*t* + 1)) are recorded at times *t* ∈ {1, 2, …, 10^6^} in a system with *N* = 200 particles and two states initially assigned with equal probabilities and homogeneous initial ages *h*_*i*_ = 0 for all *i* ∈ [*N*]. Inset: Noise strength as the standard deviation for the corresponding z values 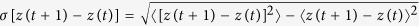.

In case *α* < 0, there are competing effects governing the dynamics of *z*. When *i* ∈ *S*^+^ convinces *j* ∈ *S*^−^, i) the set *S*^+^ gains another member who now has the youngest state increasing *z*. ii) The set *S*^−^ loses a member *j* with *τ*_*j*_ typically larger than average, making states of *S*^−^ members younger on average decreasing *z*. iii) With time advancing, all states age by the same additive rate. This makes ratios between ages smaller, driving *z* towards 1/2. In the case *α* ≪ −1, the first effect dominates. Thus, an initial advantage in *z* is amplified and the system orders quickly. For *α* ≈ −1, ordering times are large due to dominance of the second and third effects. In order to verify this idea, we numerically record data pairs (*z*(*t*), *z*(*t* + *τ*) − *z*(*t*)) with *τ* = 1. Averaging over pairs with the same or similar *z*(*t*) values, we obtain 〈*z*(*t* + *τ*) − *z*(*t*)〉 as the expected restoring force. The corresponding standard deviation is the noise strength at this *z* value. The restoring force for *z* is linear around the equilibrium point *z*(*t*) = 0.5 while the noise strength is mostly independent of *z* (see Fig. 5). This suggests to picture the dynamics around *α* = −1 as one-dimensional equilibrium in a hyperbolic potential under state-independent additive noise.

**Figure 6:**
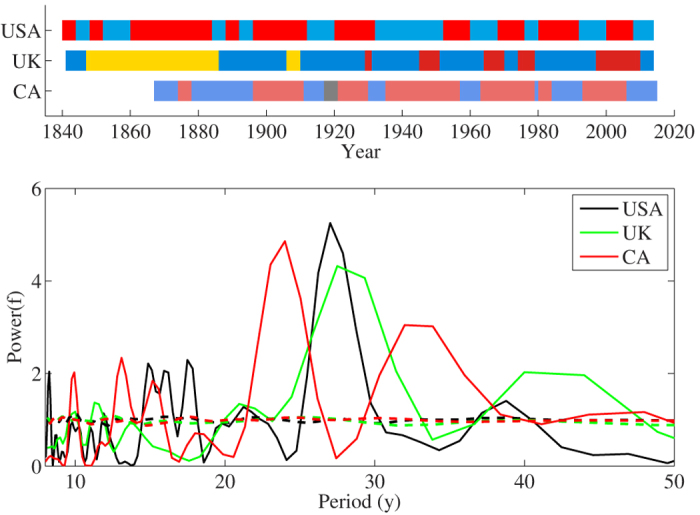
Election results for United States, United Kingdom, and Canada. Elected parties are represented by their official colors, USA: Republicans (red) and Democrats (blue); UK: Conservative (blue), Liberals (yellow), and Labour (red); Canada: Liberals (red), Unionist (gray), and Conservatives (blue). Bottom panel: Lomb -Scargle periodograms of the binary time series for each country. The Lomb-Scargle periodogram estimates the frequency spectrum of an unevenly data series based on a least squares fit of sinusoids. Dashed lines represent the level of noise as the result of shuffling the data 250 times.

Different real systems display dominance such as in the adoption of innovations^4^,^5^ and alternation as in opinion formation dynamics^47^,^48^,^49^ or economic cycles^50^. As an example, Fig. 6 shows the electoral results of the governmental elections for United States, United Kingdom, and Canada during several decades^51^. The determination of periods in the unevenly sampled time series for the governmental elections is calculated with the Lomb-Scargle method^52^,^53^. The Lomb-Scargle periodogram of the binary time series reveals the existence of alternation between the political parties, by the presence of prominent peaks well above the noise level (shuffling of the data), with periods of 20–30 years in agreement with observations^54^. This period of time coincides approximately with the length of a generation. Although our model is intended to provide support for a mechanism leading to stochastic oscillations, it could also provide information regarding the temporal scales of the process. Figure 4(c) provides the relationship between the preference of the population for young states, *α* < 0, and the frequency of the oscillation. In the range *α* ∈ [−0.2, −1] the frequency expands in the range *ω* ∈ [0.05, 0.45], that matching the period of *T* = 25 years observed in Fig. 6 allows to resolve the correspondence between the simulation Monte Carlo step and the real time to *dt* = [1.25, 11.25] years respectively. Different mechanisms have been proposed to explain political cycles: electorate disappointment^55^, voters` mood changes^54^,^56^, negativity effect^57^ or policy inertia^58^. Our study complements those mechanisms by showing that preferential dissemination of recently adopted states leads to sustained oscillations at the system level, as exhibited by the regimes *ε* < −0.39 in the model with four states and *α* < −1 in the continuous model.

## Discussion

Summarizing, we have studied the competition of states using a basic model that takes into account the aging of the state. The stability analysis of the solutions reveals the existence of two stable solutions for positive values of the persuasiveness (old state prevails) that compete for consensus. For large negative persuasiveness (young state prevails), only one solution is stable leading to oscillatory transients. We have extended our study to a more detailed continuous age model finding that, when confronting two states, the final configuration where only one state survives and the time needed to reach it is noticeably sensitive to the age of the state through the exponent *α* of the convincing probability. The continuous age model exhibits oscillations exactly as the basic model in the corresponding parameter regime, i.e. for sufficiently strong dominance of young states. Our study provides an alternative mechanism in the understanding of the dynamics of consensus formation and the observed alternation between states of different systems. Other works have reported that the increase in the persistence time yields to the appearance of an optimal period for which consensus is fastest^23^,^25^. In our model, only the preference for old opinions leads to the acceleration of the consensus time. As for the alternation between states, in the context of the spatial prisoner’s dilemma game it has also been observed that the time-dependent learning capacities lead to oscillations between cooperator and defector strategies^39^,^40^. However, the comparison of the results is not straightforward since our model takes into account the competition of states through a probability that depends on the persistence times of the interacting particles while in the mentioned references the probability of changing the state is affected by the persistence time of only one particle. It would be of interest to explore in the future the connection and implications of these two mechanisms in the ordering process and to uncover the general results from those related to the specifics of the models. Future developments should include the competition of aging states and the aging of agents in structured populations, and the presence of more than two states in the system.

## Additional Information

**How to cite this article**: Pérez, T. *et al.* Competition in the presence of aging: dominance, coexistence, and alternation between states. *Sci. Rep.* **6**, 21128; doi: 10.1038/srep21128 (2016).
